# An extremely rare case of pseudomelanosis of the urinary bladder

**DOI:** 10.1016/j.eucr.2022.102193

**Published:** 2022-08-17

**Authors:** M. Duijn, S.L. Croonen, J.A. Van der Zee

**Affiliations:** a Department of Urology, OLVG, PO Box 95500, 1090 HM, Amsterdam, the Netherlands; b Department of Pathology, OLVG, PO Box 95500, 1090 HM, Amsterdam, the Netherlands

**Keywords:** Pseudomelanosis, Melanosis, Pigmentation, Melanin, Melanin-like pigment, Urinary bladder

## Abstract

Pseudomelanosis (PM) is a rare, benign, condition that is characterized by deposition of melanin and/or melanin-like pigment in mucosal cells and macrophages and is best known as the entity pseudomelanosis coli.

Pseudomelanosis primary of the urinary bladder has been reported only in a handful of cases worldwide. This article reports an extremely rare case of pseudomelanosis of the urinary bladder in a 79-year-old male with a history of macroscopic painless hematuria.

## 1 Introduction

Pseudomelanosis (PM) is a rare, benign, condition that is characterized by the presence of black and brown pigmentation in mucosal cells and macrophages due to deposition of iron, melanin or melanin-like pigment. It is best known as the entity pseudomelanosis coli, and also described in the stomach, duodenum and jejunum.^1^ The precise etiology and pathogenesis are largely unknown.^2^ However, it is thought to be linked to the use of specific types of medications and chronic diseases, including iron supplements, beta-blockers, thiazide diuretics, diabetes mellitus, hypertension and renal diseases.^3^ We describe an extremely rare case of pseudomelanosis of the bladder in a 79-year-old male.

## 2 Case presentation

**Fig. 1:**
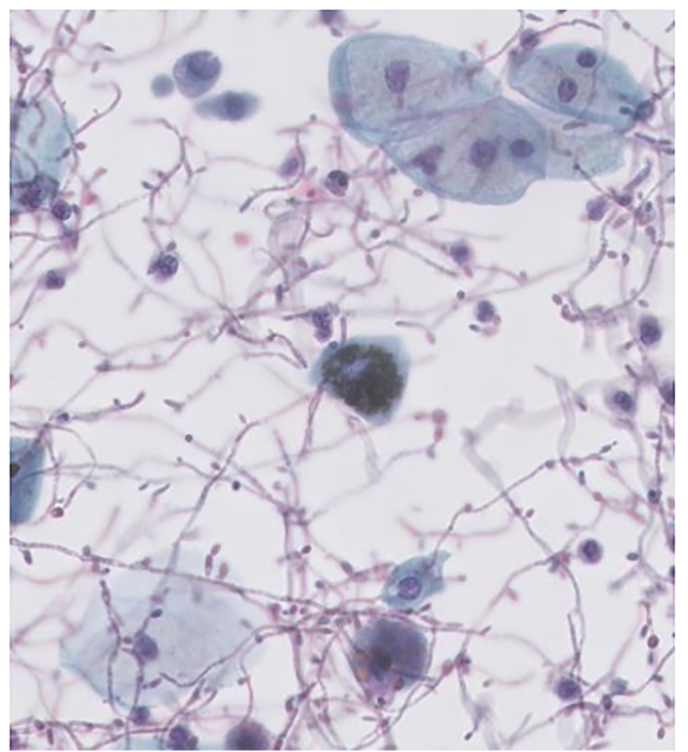
Cytology with candida and a pigmented urothelial cell.

A 79-year-old male presented with a seven weeks history of macroscopic painless hematuria. Lower urinary tract symptoms (LUTS) were absent, especially stranguria and pollakiuria. His past medical history included cataract and left-sided adrenalectomy. He took medication regularly, including amlodipine, spironolactone and rivaroxaban. There were no risk factors for urothelial carcinoma. Physical examination did not reveal any abnormalities. Urine analysis was negative for nitrite and leukocytes. Microscopic examination of urine sediment showed the presence of erythrocytes, dysmorphic erythrocytes were not found. Urine cytology revealed a severe candida infection with several pigment-laden cells, consistent with urothelial cells with melanin pigment (Fig. 1). Cystoscopy showed trabeculation of the bladder, diverticula, an enlarged median prostatic lobe and a solid mass, 1–2cm, on the right posterior wall was suggested. However, the vision was poor due to debris. An CTU of the kidneys and upper urinary tract did not demonstrate any abnormalities.

**Fig. 2:**
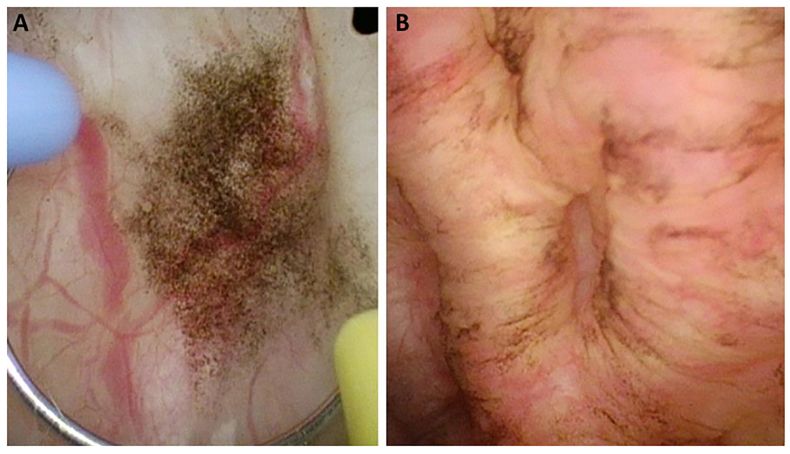
A and B. Multiple black and brown pigmented lesions in the bladder during surgery. (For interpretation of the references to colour in this figure legend, the reader is referred to the Web version of this article.)

After consent, the patient underwent surgery through a transurethral approach. A tumor of the bladder was not seen. However, multiple black and brown pigmented lesions were found and biopsied separately (Fig. 2A and B).

**Fig. 3:**
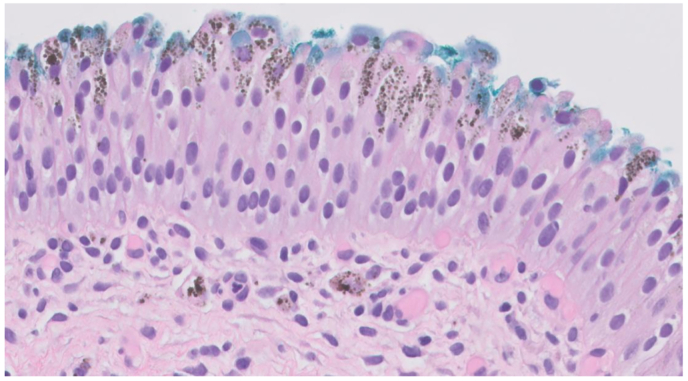
Histology with abundant pigment in the upper layers of the urothelium and several pigment laden macrophages in the lamina propria.

The histologic specimen consisted of urothelium with a normal architecture. The upper layers of the urothelium contained cytoplasmic melanin pigment, which was confirmed using a Schmorl stain. No melanocytes were found. The lamina propria contained several pigment-laden macrophages (Fig. 3).

Per-operative a three-way Foley catheter with continuous irrigation system was inserted. The catheter was removed on the first day postoperative. Because of increasing post-void residual urine (>450 cc) the patient was discharged with a two-way indwelling catheter and an alpha-1-blocker was prescribed. After one week the patient was unable to adequately empty his bladder without catheter, clean intermittent self-catheterization (CISC) was educated. The hematuria resolved without further treatment. He will be followed up with cystoscopy 6–8 months later.

## 3 Discussion

Pseudomelanosis is a rare condition of deposition of melanin and/or melanin-like pigment in mucosal cells and macrophages and is best known as the entity pseudomelanosis coli. Pseudomelanosis primary of the bladder or urinary tract is extremely rare, only a handful of cases been reported worldwide. It was first described in 2006 by Wieringa et al. this was also at our institution.^4^

The precise etiology is unknown, though there may be an association with the use of specific categories of medications and chronic diseases, including iron supplements, beta-blockers, thiazide diuretics, diabetes mellitus, hypertension and renal diseases.^2^^,^^3^ Histological examination is essential in diagnosing the condition. The presence of melanin can be confirmed by Schmorl staining.

Pseudomelanosis, in most cases asymptomatic, is often found by accident during cystoscopy or surgery and is on itself no indication for treatment. In our case the patient presented with hematuria, most likely based on candida cystitis as there were no other urinary tract abnormalities found during surgery. However, he had no LUTS and the hematuria resolved after one week without further treatment.

Although considered benign, the rarity of the lesions warrants regular cystoscopic evaluation and biopsy to assess the risk of malignant transformation, pseudomelanosis has been described in association with malignant melanoma and urothelial carcinoma.^5^ Clinical guidelines regarding the length of follow up are absent. Our patient will be followed up with cystoscopy in 6–8 months.

Postoperative the patient showed elevated post voiding residual volumes after removal of the three-way Foley catheter. Re-catheterization was necessary and medical treatment by an alpha-1-blocker monotherapy was started. After one week he failed to void successfully without catheter and CISC was taught. During cystoscopy an enlarged median prostatic lobe was seen. Thereby, it is most likely that the impaired bladder emptying is based on enlargement of the prostate in the context of benign prostatic hyperplasia.

## 4 Conclusion

Pseudomelanosis of the bladder is an extremely rare entity with only a few cases reported worldwide. Histopathological examination is essential in diagnosing this condition. Although considered benign, follow up with cystoscopy is recommended by several authors.

## Consent

Informed consent was obtained from the patient for publication of this case report in accordance with the journals patient consent policy.

## Funding statement

No funding to declare.

## Ethical approval

The study conforms to recognized standards of World Medical Association Declaration of Helsinki.

## Author contributions

**M. Duijn:** Conceptualization, Methodology, Writing - original draft preparation, Resources. **S.L. Croonen:** Investigation, Visualization, Writing - review and editing. **J.A. Van der Zee:** Writing - review and editing, Validation, Supervision.

## Data availability

The data used and/or analyzed ruing the current study are available from the corresponding authors per request.

## Declaration of competing interest

The authors declare no conflict of interest.
